# Effect of beam profile measurement on arc therapy plan quality assurance: a case study

**DOI:** 10.1002/acm2.12071

**Published:** 2017-03-29

**Authors:** Leonard H. Kim, Irina Malajovich, Meral L. Reyhan, Jinyu Xue, Joo Han Park

**Affiliations:** ^1^ Department of Radiation Oncology MD Anderson Cancer Center at Cooper 2 Cooper Plaza Camden NJ 08103 USA

**Keywords:** arc therapy, beam modeling, quality assurance

## Abstract

We present an example when profile measurement and modeling of an Elekta Agility multileaf collimator (MLC) had a large effect specifically on arc therapy plan quality assurance (QA) results using ArcCheck. ArcCheck absolute dose measurements of these plans were systematically lower than planned by 3–10%. Failing QA results were seen even with unmodulated static and conformal arcs. Furthermore, the effect was found to be dependent on collimator angle, with worse results associated with near‐zero collimator angles. In contrast, step‐and‐shoot QA results were not affected. Changing the beam model to match steeper profile measurements obtained using a different measurement device resolved the problem. This case study demonstrates that conventional gamma index analysis can be sensitive to small profile modeling changes.

## 1 Introduction

Our department recently switched to the Pinnacle^3^ (Philips Healthcare, Fitchburg, WI, USA) treatment planning system (TPS) at two facilities. The first facility has a TrueBeam (Varian Medical Systems, Palo Alto, CA, USA). The second facility has two Infinities (Elekta AB, Stockholm, Sweden) with the Agility multileaf collimator (MLC). Introduction of volumetric modulated arc therapy (VMAT) using 6 MV photons was planned at both locations, and new beam measurements were acquired for modeling. After the TPS change at our Varian site, patient‐specific VMAT quality assurance (QA) using ArcCheck (Sun Nuclear Corporation, Melbourne, FL, USA) typically gave gamma index pass percentages in the high 90 s at 3%/3 mm tolerances in absolute dose mode using a 10% low‐dose threshold.

At our Elekta site, however, testing showed VMAT QA gamma index pass percentages typically in the 45–85% range. These poor results were attributable to ArcCheck absolute dose measurements being systematically below Pinnacle planned doses (3–10% lower depending on the plan), although relative dose distributions agreed well. The low measured doses were seen only with arc deliveries and were observed for unmodulated conformal and static arcs as well as VMAT. In contrast, 10 step‐and‐shoot IMRT plans delivered to the ArcCheck yielded acceptable pass rates (93 ± 3%, mean ± 1 SD). Ion chamber measurements at the center of the ArcCheck confirmed that delivered doses for arcs were lower than planned (1–6% lower), though not to the same degree as the ArcCheck measurements (3–10% lower).

**Figure 1:**
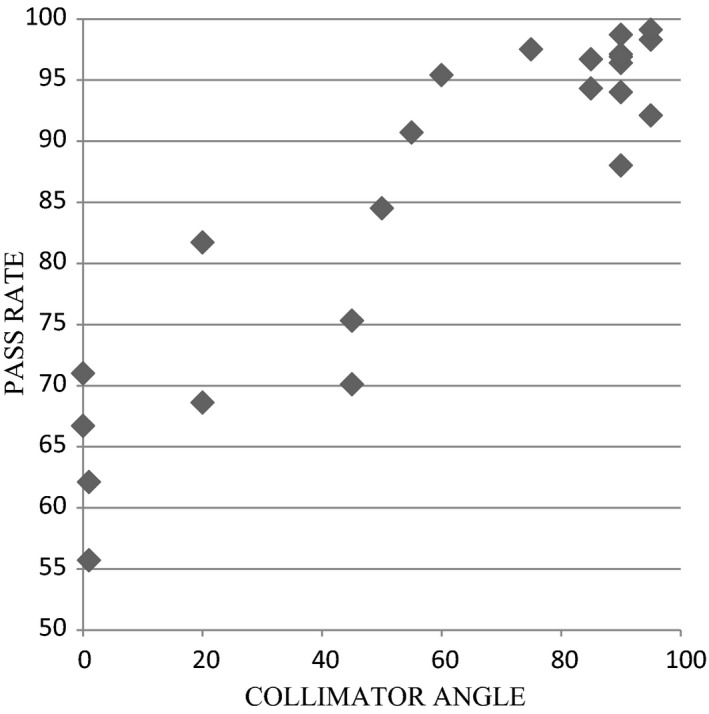
Arc delivery QA gamma index pass % vs. collimator angle. Data points include VMAT head‐and‐neck, VMAT prostate, and conformal prostate.

Notably, QA pass percentages were correlated with collimator angle. Arcs with collimator angle near 0 (IEC 61217 scales, i.e., leaf motion parallel to gantry motion) exhibited worse pass percentages and larger dose differences than arcs with collimator angle near 90 degrees, where pass rates generally satisfied the 90% standard (Fig. 1). This dependence on collimator angle and the fact that poor QA results were limited to arc therapies led to suspicion of a machine issue such as gravity and/or gantry motion affecting the MLC. However, vendor testing of the MLC and other components could not identify any problem. Testing using our old TPS, Monaco (Elekta AB), did not reproduce the poor QA results, further eliminating equipment issues as the culprit.

**Figure 2:**
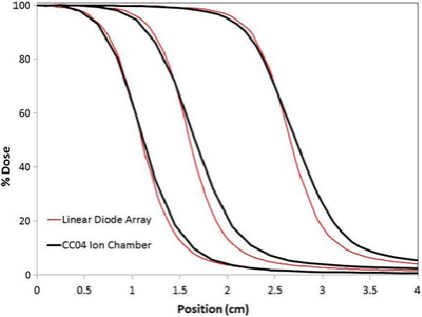
Profiles for 2 × 2, 3 × 3, and 5 × 5 fields acquired with LDA and CC04 ion chamber.

It was noticed that the profiles of the Monaco beam model were steeper than those of the Pinnacle model. Small‐field profile measurements for Monaco had been made with a Linear Diode Array (LDA, IBA Dosimetry, Schwarzenbruck, Germany), while a CC04 chamber (IBA Dosimetry) was used for the Pinnacle measurements (Fig. 2). We speculate the difference in profiles obtained with the two instruments was due to different detector sizes contributing to different dose volume averaging.^1^ The steeper profiles obtained with the LDA were considered closer to “reality”, and we decided to partially remodel the beam on the basis of this older data.

## 2 Methods

We partially remodeled our beam to match the penumbra and tails measured using the LDA. Only the six “Out of Field” parameters were modified. Initially, the MLC “Offset Calibration” table was modified as well to match the LDA data, as the field edges appeared shifted ~0.2 mm outward relative to the CC04 data. However, the LDA profiles had been acquired along the beam axes, i.e., at the interleaf gap rather than the middle of the leaf, resulting in the apparent field size increase. Radiochromic film measurements of the MLC field edge confirmed a 0.2 mm difference between leaf center and leaf gap. As the vendor recommendation is to measure at leaf center, we ultimately chose not to modify the MLC offset calibration table to match the LDA field sizes.

## 3 Results and discussion

**Figure 3:**
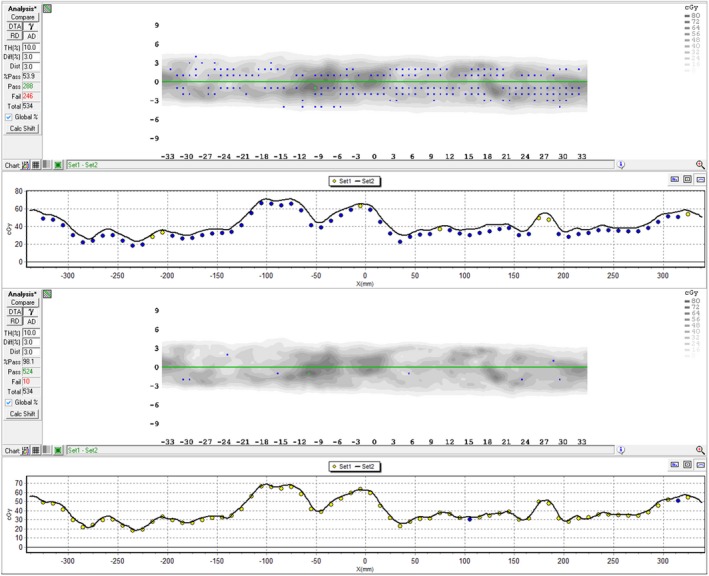
Sample absolute dose comparison between VMAT delivered and calculated dose using the initial (Top) and new (Bottom) beam models. For this plan, the new model pass % is 98.1% vs 53.9% using the old model. Without a 0.2 mm MLC offset adjustment to account for profiles not acquired at leaf center, the new model pass rate would have been 92%.

ArcCheck results for VMAT using the revised model showed excellent agreement between TPS and measurement (Fig. 3), while step‐and‐shoot IMRT results remained as good or better compared to the original model. Prior to remodeling, our mean pass rate was 88 ± 11% (1 SD) for 27 VMAT plans. Most of these plans had a collimator angle of 90°, which had been identified as yielding the highest pass rates. After remodeling, our mean pass rate for 67 VMAT plans was 98 ± 1% with no restriction on collimator angle. Over a 5‐month investigation, with vendor support, we had explored many possible causes but had been slow to suspect our beam model for a few reasons. Our model had matched our measured data well, and validation tests for static fields all the way to complex step‐and‐shoot deliveries had all shown excellent agreement. We were hard‐pressed to explain, on beam modeling grounds, the observed collimator angle effect and the poor results from even barely modulated arcs. Also, the same measurement devices and modeling techniques had been used in a successful implementation of VMAT at another facility. We do not know why we did not encounter the same issue when modeling our Varian machine. We speculate the difference could be because of the use of both jaw‐ and MLC‐shaped fields when modeling the Varian. The Varian has both x‐ and y‐jaws, and its MLC is a tertiary collimator. In contrast, the Elekta MLC is a secondary collimator, and there is no x‐jaw.

**Figure 4:**
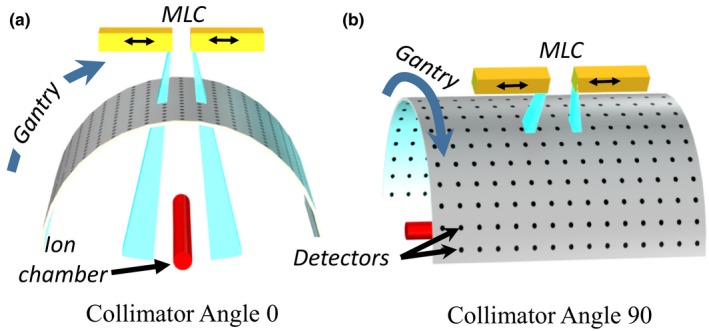
Representation of different collimator and detector geometries. (a) For arc therapy, if the MLC is parallel to the gantry rotation, an ArcCheck detector will see the integrated dose profile of the leaf tips, even if the leaves are not moving. For a central ion chamber or for collimator angle 90° (b), integrated leaf tip dose will be measured only if a leaf tip travels over the detector.

How can profile measurement and modeling manifest only in arc therapies at certain collimator angles? At near‐zero collimator angle, when leaves are oriented along the gantry motion, for any individual ArcCheck detector, dose from the entire penumbra and tail of a leaf tip is seen and integrated at least twice as the leaf gap sweeps across the detector whether the leaves themselves are moving or not (Fig. 4a). Particularly for the small leaf separations characteristic of VMAT, this can result in a systematic overestimation of dose by the TPS if the modeled penumbra and tail are too wide. For example, with the same central dose, the integrated dose difference for a 1 × 1 field was 9% between our two models.

Even for a static arc, where the MLC leaves are not moving, this effect is seen because of the position of the ArcCheck's diode detectors, which are arranged on a cylinder of radius 10.4 cm around the gantry axis. In contrast, for a centrally located ion chamber, the fully‐integrated penumbra and tail dose will be seen only if the central leaves dynamically travel over the central axis, leading to our observation that a dose discrepancy may be seen at isocenter, but not of the same magnitude as seen by the more peripheral ArcCheck detectors. For arcs with collimator angle close to 90, leaf motion is perpendicular to gantry motion. Either the side or the tip of leaves may or may not partially or completely sweep a detector, mitigating any consistent, systematic dose difference (Fig. 4b). For step‐and‐shoot deliveries, a detector will only see penumbra and tail dose if a leaf tip happens to coincide with it, and even then at only one point along the dose profile.

Bedford et al. have reported low measured dose during VMAT verification, though to a much smaller degree (1–2%) than we initially observed.^2^ They attributed this to limitations in the applicability of equivalent square‐based output factors as well as scatter calculation accuracy. Contrary to their experience, we were able to influence our results through modeling. Due to different measurement and QA devices used, direct comparison of our experience with Bedford et al. and to that reported by Saenz et al.^3^ is difficult. However, an examination of their models as well as other institutional models suggests there remains considerable variability in both the measurement and modeling of the Agility MLC.

## 4 Conclusion

Small differences in beam profile measurement and modeling can have large effects on VMAT QA results under certain, perhaps unexpected, conditions. Conventional gamma index analysis appears sensitive to small differences in profile modeling.

## Acknowledgment

We thank Geoffrey Ibbott for reading through the manuscript and for his suggestions.

## Conflict of interest

There are no conflicts of interest.
